# A NEW DATA SET TO EXAMINE HOME HEALTH AGENCIES

**DOI:** 10.1093/geroni/igae098.2791

**Published:** 2024-12-31

**Authors:** Lacey Loomer, Sweta Patel, Emmanuelle Belanger, Pedro Gozalo

**Affiliations:** University of Minnesota Duluth, Duluth, Minnesota, United States; Brown University, Providence, Rhode Island, United States; Brown University, Providence, Rhode Island, United States; Brown University, Providence, Rhode Island, United States

## Abstract

Using Outcome and Assessment Information Set (OASIS) data from 2015 to 2019, we developed a dataset representing home health agency Medicare patients that will be made publicly available for researchers and policymakers to compare over time and geographically. We created unique home health stays based on start of care and discharge assessments from OASIS and aggregated the data to the calendar year, agency, county, state, insurance (fee-for-service vs. Medicare Advantage), and by admission (institutional vs. community). We identified demographics for home health stays using the Medicare Beneficiary Summary File. We characterize the home health stays using OASIS variables including length of stay, activities of daily living, instrumental activity of daily living, cognitive function, common diagnoses, urinary tract infections, incontinence, history of falls, and pressure ulcers. Type of admission was determined based on Medicare claims for hospital, nursing home, or inpatient rehabilitation facility in the fourteen days prior to home health admission. Insurance was determined using the Medicare Beneficiary Summary File identified in each month of the home health stay, if a patient changed insurance through the stay, the stay’s attribution was prorated by month to each type of health insurance. There were 6,761,470 home health stays from 5,051,631 Medicare beneficiaries in 2016 compared to 6,703,521 home health stays from 4,969,010 Medicare beneficiaries in 2019. In 2019, 1994 home health agencies served fee-for-service patients only, 94 served Medicare Advantage patients only, and 8,720 served both.

